# 

**Published:** 1898-09

**Authors:** 


*The following donations have been received since the publication of the list in June:*

*August 31st, 1898.*

| | |
|---|---|
| American Ophthalmological Society (1) ... ... | 20 volumes. |
| College of Physicians of Philadelphia (2) ... ... | 13 ,, |
| L. M. Griffiths (3) ... ... ... ... ... ... ... | 1 volume. |
| Official Board of Advertising for the Isle of Man (4) | 1 ,, |
| Medical Society of London (5) ... ... ... ... | 10 volumes. |
| The Family of the late J. S. Metford (6) ... ... | 27 ,, |
| J. E. Shaw, M.B. (7) ... ... ... ... ... ... ... ... | 22 ,, |
| R. Shingleton Smith, M.D. (8) ... ... ... ... ... | 3 ,, |

Unbound periodicals have been received from the Liverpool Medical Institution and from the Family of the late Mr. J. S. Metford. Unframed pictures have been presented by the Arlington Chemical Company, the Editor of *The Boston Medical and Surgical Journal*, Dr. George M. Gould, Dr. J. H. Hunt, Dr. S. Weir Mitchell, the Editor of *The Railway Surgeon*, and Dr. J. O. Symes.

### TWENTY-NINTH LIST OF BOOKS.

The titles of books mentioned in previous lists are not repeated.

The figures in brackets refer to the figures after the names of the donors, and show by whom the volumes were presented. The books to which no such figures are attached have either been bought from the Library Fund or received through the *Journal*.

*Baby Feeding* ... ... ... ... ... ... ... ... ... ... ... ... ... ... 1898

**Bangs and W. A. Hardaway, L. B.** [Eds.] *An American Text-Book of Genito-Urinary Diseases, Syphilis, and Diseases of the Skin.* One volume in two ... ... ... ... 1898

**Barker, F. H.** ... *On the Hygienic Management of Infants and Children* (6) 1859

**Biedermann, W.** ... *Electro-Physiology.* (Tr. by Frances A. Welby) 2 vols. 1896-98

**Bird, G.** ... ... *Urinary Deposits* ... ... ... ... ... ... (6) 4th Ed. 1853

**Brown, J. D.** ... *Library Classification* ... ... ... ... ... ... ... 1898

**Burgoyne, F. J.** ... *Library Construction* ... ... ... ... ... ... ... ... 1897

**Carless, W. Rose and A.** *A Manual of Surgery* ... ... ... ... ... ... 1898

**Carpenter, W. B.** *Principles of Physiology, General and Comparative.* (6) 3rd. Ed. 1851

*Chambers's Encyclopædia.* 10 vols. ... ... ... ... ... ... ... ...New Ed. 1895

**Chreiman, M. A.** *Health Loss and Gain* ... ... ... ... ... ... ... 1898

**Churchill, F.** ... *Diseases of Females* ... ... ... ... ... ... ... (6) 1838

**Cooke, T. and F. G. H.** *Tablets of Anatomy.* 3 parts. ... [11th Ed.] 1898
**Corfield, W. H.** ... *Dwelling Houses* ... ... ... ... ... ... 4th Ed. 1898
**Cory, R.** ... ... *Theory and Practice of Vaccination*... ... ... ... ... 1898
**Down, J. L.** ... *Mental Affections of Childhood and Youth* ... ... ... 1887
*Edinburgh and Neighbourhood, Pocket Guide to* ... ... ... ... ... (8) 1898
**Edkins, E. Klein and J. S.** *Elements of Histology* ... ... [3rd Ed.] 1898
**Fenwick, S.** ... *Obscure Diseases of the Abdomen* ... ... ... ... ... 1889
**Forster, J. C.** ... *The Surgical Diseases of Children* ... .. ... ... (6) 1860
**Fürbringer, P.** ... *Diseases of the Kidneys and Urinary Organs.* (Tr. by W. H. Gilbert) Vol. II.... ... ... ... .. 1898
**Gregory, W.** ... *Outlines of Chemistry.* Part II.—Organic Chemistry (6) 1845
**Hancock, H.** ... *On the Operative Surgery of the Foot and Ankle-Joint*... 1873
**Hardaway, L. B. Bangs and W. A.** [Eds.] *An American Text-Book of Genito-Urinary Diseases, Syphilis, and Diseases of the Skin.* One volume in two ... ... ... 1898
**Hassall, A. H.** ... *Adulterations detected in Food and Medicine* ... ... (6) 1857
**Hoblyn, R. D.** ... *A Dictionary of Terms used in Medicine* (6) 2nd Ed. 1844
**Holmes, T.**... ... *Sir Benjamin Collins Brodie* ... ... ... ... ... ... 1898
**Hooper, [R.]** ... *Physician's Vade-Mecum.* 6th Ed. by W. A. Guy (6) 1858
**Ireland, W. W.** *Mental Affections of Children* ... ... ... ... ... ... 1898
*Isle of Man, Official Guide to the* ... ... ... ... ... ... ... ... ... (4) 1898
**Jakob, C.** ... ... *Atlas of Clinical Diagnosis and Internal Diseases.* (Ed. by A. A. Eshner) ... ... ... ... ... 1898
**Jellett, H.**... ... *A Short Practice of Midwifery* ... ... ... ... ... 1897
**Kelly, H. A.** ... *Operative Gynecology.* Vol. II. ... ... ... ... ... 1898
**Kelynack, T. N.** *Renal Growths* ... ... ... ... ... ... ... ... ... ... 1898
**Klein and J. S. Edkins, E.** *Elements of Histology* ... ... [3rd Ed.] 1898
**Lazarus-Barlow, W. S.** *A Manual of General Pathology* ... ... ... 1898
*Lewis's Library Catalogue.* ... ... ... ... ... ... ... ... ... New Ed. 1898
*Lippincott's Medical Dictionary* ... ... ... ... ... ... ... ... ... .. ... 1897
**Loomis and W. G. Thompson, A. L.** [Eds.] *A System of Practical Medicine.* Vol. III. ... ... ... ... ... ... ... ... ... 1898
**Lyman, H. M.** ... *Artificial Anæsthesia and Anæsthetics* ... ... ... 1882
**Manders, H.** ... *The Ferment Treatment of Cancer and Tuberculosis* ... 1898
**Manson, P.** ... *Tropical Diseases* ... ... ... ... ... ... ... ... 1898
**Martin, J. W. White and E.** *Genito-Urinary Surgery and Venereal Diseases* 1898
**Martindale and W. W. Westcott, W.** *The Extra Pharmacopœia* 9th Ed. 1898
**Massey, G. B.** ... *Conservative Gynecology and Electro-Therapeutics* 3rd Ed. 1898
**Meredith, J.** ... *On the Vomiting of Pregnancy and Menorrhagia* ... (3) 1888
**Norris and C. A. Oliver, W. F.** [Eds.] *System of Diseases of the Eye.* Vol. III. [1898]
**Oliver, W. F. Norris and C. A.** [Eds.] *System of Diseases of the Eye.* Vol. III. [1898]
**Osler, W.** ... ... *The Cerebral Palsies of Children* ... ... .. ... ... 1889
*Pathology, An Atlas of Illustrations of.* (New Syd. Soc.) Fasc. XI. ... 1898
**Penrose, C. B.** ... *Text-Book of Diseases of Women* ... ... 2nd. Ed. 1898
*Pharmacopœia, British, 1898. Pocket Notes on the New Pharmacopœia* ... 1898
*The British Pharmacopœia, 1898. Additions, Omissions and Alterations* ... .. ... ... ... ... 6th Ed. 1898
*Review of the New British Pharmacopœia, 1898, with Notes of Alterations and Additions* ... ... ... [1898]
**Pick, A.** ... ... *Pathologie und pathologischen Anatomie des Centralnervensystems* ... ... ... ... ... ... ... 1898

**Pollard, J.** ... ... *The Care of Public Health and the New Fever Hospital in Edinburgh* ... ... ... ... ... ... ... ... (8) 1898

**Pratt, A.** ... ... *Poisonous, Noxious, and Suspected Plants* ... ... (6) [N.D.]

**Richardson, R.** ... *On the Nature of Life* ... ... ... ... (6) 2nd Ed. 1879

**Rideal, S.** ... ... *Practical Organic Chemistry.* ... ... ... ... 2nd Ed. 1898

**Rose and A. Carless, W.** *A Manual of Surgery.* ... ... ... ... ... ... ... 1898

**Ruddock, E. H.** *The Homœopathic Vade Mecum of Modern Medicine and Surgery* ... ... ... ... ... ... (6) 4th Ed. 1871

**Ruschenberger, W. S. W.** *An Account of the College of Physicians of Philadelphia* ... ... ... ... ... ... ... ... (2) 1887

**Saunders, W. S.** ... *Report on the Sanitary Condition of the City of London for 1897* ... ... ... ... ... ... ... ... (8) 1898

**Simon, Sir J.** ... *English Sanitary Institutions* ... ... ... ... 2nd Ed. 1897

**Simpson, J. Y.** ... *Homœopathy: its Tenets and Tendencies* (6) 3rd Ed. 1853

**Smith, R. W.** ... *A Treatise on Neuroma.* (Fasc. XI. of N.S.S. *Atlas of Pathology*) ... ... ... ... ... ... ... ... ... 1898

**Smith, W. T.** ... *A Manual of Obstetrics* ... ... ... ... ... ... (6) 1858

**Solly, S. E.** ... ... *Medical Climatology* ... ... ... ... ... ... ... ... 1897

**Squire, P.** ... ... *Companion to the British Pharmacopœia* (6) 7th Ed. 1869

**Stokes, Sir W.** ... *William Stokes* ... ... ... ... ... ... ... ... 1898

**Tanner, T. H.** ... *Practice of Medicine* ... ... ... ... ... ... (6) 4th Ed. 1861

**Thin, G.** ... ... *Psilosis* ... ... ... ... ... ... ... ... ... 2nd Ed. 1897

**Thompson, A. L. Loomis and W. G.** [Eds.] *A System of Practical Medicine.* Vol. III. 1898

**Thomson, A. T.** ... *The London Dispensatory* ... ... ... (6) 9th Ed. 1837

**Thomson, J.** ... *Clinical Examination and Treatment of Sick Children* ... 1898

**Toynbee, J.** ... *The Diseases of the Ear* ... ... ... ... ... ... ... (6) 1868

**Turnbull, L.** ... *Manual of Anæsthetics* ... ... ... ... ... 2nd Ed. 1880

**Walker, G. A.** ... *A Practical Chart of Diseases of the Skin* ... ... (6) [N.D.]

**Walsham, W. J.** *Nasal Obstruction* ... ... ... ... ... ... ... ... ... 1898

**Weber, H. and F. P.** *The Mineral Waters and Health Resorts of Europe*... 1898

**Westcott, W. Martindale and W. W.** *The Extra Pharmacopœia* 9th Ed. 1898

**White and E. Martin, J. W.** *Genito-Urinary Surgery and Venereal Diseases* 1898

**White, W. H.** ... *Materia Medica* ... ... ... ... ... ... ... 3rd Ed. 1898

**Williams, D.** ... *Medical Diseases of Infancy and Childhood* ... ... ... 1898

**Wilson, E. M.** ... *Notes on Malaria in connection with Meteorological Conditions in Sierra Leone*... ... ... ... 2nd Ed. 1898

---

### TRANSACTIONS, REPORTS, JOURNALS, &c.

American Journal of Obstetrics, The ... ... ... ... ... Vol. XXXVII. 1898

American Journal of the Medical Sciences, The ... ... Vol. CXV. 1898

American Ophthalmological Society, Transactions of the (1) Vols. II., Part I.—VII., Part I. 20 parts 1873–94

American Pediatric Society, Transactions of the ... ... Vol. IX. 1897

Boston Medical and Surgical Journal, The ... ... Vol. CXXXVIII. 1898

Bristol Health Report, 1897 ... ... ... ... ... ... ... ... ... ... ... 1898

British Gynæcological Journal, The ... ... ... ... ... Vol. XIII. 1897

British Medical Journal, The ... ... ... ... ... ... Vol. I. for 1898

| | | |
|---|---|---|
| Dublin Journal of Medical Science, The ... ... ... ... | Vol. CV. | 1898 |
| Edinburgh Medical Journal, The ... ... ... ... ... | N.S., Vol. III. | 1898 |
| Glasgow Medical Journal, The ... ... ... ... ... ... | Vol. XLIX. | 1898 |
| Hospitals— | | |
| King's College Hospital Reports ... ... ... ... ... | Vol. IV. | 1898 |
| Saint Bartholomew's Hospital Reports ... ... | Vol. XXXIII. | 1898 |
| Recherches sur l'Épilepsie et l'Idiotie (Bicêtre) | Vol. XVIII. | 1898 |
| Hospitals and Charities, Burdett's ... ... ... ... ... ... ... ... | | 1898 |
| International Congress of Dermatology, Transactions of the Third, 1896. | | 1898 |
| Journal of the American Medical Association, The ... ... | Vol. XXX. | 1898 |
| Lancet, The ... ... ... ... ... ... ... ... ... ... ... | Vol. I. for | 1898 |
| Medical Chronicle, The ... ... ... ... ... ... | N.S., Vol. VIII. | 1897–98 |
| Medical News, The ... ... ... ... ... ... ... ... ... | Vol. LXXII. | 1898 |
| Medical Press and Circular, The ... ... ... ... ... | [Vol. CXVI.] | 1898 |
| Medical Society of London, Transactions of the (5) | [N.S.], Vol. II. | 1862 |
| " " " Proceedings of the (5) Vols. I.—V., 1872–81, VII.—IX., 1884–86, XV., | | 1892 |
| New York Medical Journal, The ... ... ... ... ... ... | Vol. LXVII. | 1898 |
| Obstetrical Society of London, Transactions of the ... | Vol. XXXIX. | 1898 |
| Philadelphia, Transactions of the College of Physicians of (2) 3rd Ser., Vols. I.—IX., 1875–87, XIII.—XV., | | 1891–93 |
| Practitioner, The ... ... ... ... ... ... ... ... ... ... | Vol. LX. | 1898 |
| Progrès médical, Le ... ... ... ... ... ... ... ... | 3e Sér., Tome VII. | 1898 |
| Retrospect of Medicine, Braithwaites's ... ... ... ... | Vol. CXVII. | 1898 |
| Sanitary Record, The ... ... ... ... ... ... ... ... ... | Vol. XXI. | 1898 |
| Year-Book of Scientific and Learned Societies ... ... ... ... ... ... | | 1898 |

---

On p. 188, line 3 from bottom, for "1889" read "1898."

---

### Local Medical Notes.

---

### BRISTOL.

THE SEWAGE.—*The Sanitary Record* says that "for some months past the question of dealing with the sewage of Bristol has been occupying the attention of a Sub-committee of the Bristol Sanitary Authority, and the advice has been obtained of Mr. Santo Crimp, of the firm of Messrs. Tayler, Sons, and Santo Crimp, engineers, of Westminster. Several meetings have been held, and the Sub-committee has received Mr. Santo Crimp's report, which will probably be recommended to the full Committee for adoption at an early date. The scheme, we understand, is of a very comprehensive character, dealing with the whole question of sewage disposal, and freeing the river Avon from all pollution."

### *PUBLICATIONS RECEIVED.*

From FÉLIX ALCAN, Paris:

*Recherches sur l'Épilepsie et l'Idiotie.* Vol. XVIII. 1898.

*Onzième Congrès de Chirurgie—Procès-Verbaux, Mémoires et Discussions.* 1897.

From BAILLIÈRE, TINDALL AND COX, London:

*Nasal Obstruction.* By W. J. WALSHAM, M.B., C.M. 1898.
*Theory and Practice of Vaccination.* By ROBERT CORY, M.A., M.D. 1898.
*A Manual of Surgery.* By WILLIAM ROSE, M.B., B.S., and ALBERT CARLESS, M.S. 1898.

From JOHN BALE, SONS & DANIELSSON, LTD., London:

*The Journal of Balneology and Climatology.* July, 1898.

From FRIEDR. BAYER & CO., Elberfeld:

*Clinical Excerpts.* June—August, 1898.

From GEORGE BELL & SONS, London:

*The Animals' Friend.* July, 1898.

From BENNETT BROTHERS LD., Bristol:

1897. *City & County of Bristol. Annual Report of the Medical Officer of Health.* 1898.

From CALIFORNIA MEDICAL COLLEGE, San Francisco:

*California Medical Journal.* June—August, 1898.

From CASSELL AND COMPANY, LIMITED, London:

*Elements of Histology.* By E. KLEIN, M.D., F.R.S., and J. S. EDKINS, M.A., M.B. Revised and Enlarged Edition. 1898.
*Tropical Diseases.* By PATRICK MANSON, M.D., LL.D. 1898.
*Medical Diseases of Infancy and Childhood.* By DAWSON WILLIAMS, M.D. 1898.

From J. & A. CHURCHILL, London:

*Materia Medica.* By W. HALE WHITE, M.D. Third Edition. 1898.
*A Short Practice of Midwifery.* By HENRY JELLETT, B.A., M.D., B.Ch., B.A.O. 1897.
*A Manual of General Pathology.* By WALTER SYDNEY LAZARUS-BARLOW, B.A., B.C., M.D. 1898.
*Psilosis.* By GEORGE THIN, M.D. Second Edition. 1897.

From CHURCH MISSIONARY SOCIETY, London:

*Mercy and Truth.* July—September, 1898.
"*Half and Half.*" By Miss E. F. FOX. [N.D.]

From WILLIAM F. CLAY, Edinburgh:

*Clinical Examination and Treatment of Sick Children.* By JOHN THOMSON, M.D. 1898.

From THE F. A. DAVIS COMPANY, Philadelphia:

*Conservative Gynecology and Electro-Therapeutics.* By G. BETTON MASSEY, M.D. Third Edition. 1898.

From EDUARDO DUBLAN, Mexico:

*Boletin del Consejo superior de Salubridad.* Mayo 31—Julio 31, 1898.

From Dr. LANDON B. EDWARDS, Richmond, U.S.A.:

*The Virginia Medical Semi-Monthly.* May 27—July 22, 1898.

From EVANS, GADD & CO., Exeter:

*B.P. Synopsis, 1898.* Compiled by H. WIPPELL GADD. [Third Edition.] 1898.

From Dr. L. WEBSTER FOX, Philadelphia:

*Injuries of the Eyelids and Eyeballs.* By L. WEBSTER FOX, A.M., M.D. 1897.
*Cataract Operations; Mules's Operation Illustrated by Skiagraphs; Capsulotomy; Operation for Pterygium.* By L. WEBSTER FOX, A M., M.D. [1898.] [Reprint.]

From 3,509 Franklin Avenue, St. Louis:

*The Tri-State Medical Journal and Practitioner.* May, 1898.

From GIORNALE INTERNAZIONALE DI MEDICINA PRATICA, Naples:

*Nuove ricerche sull'azione della diuretina (Knoll) nelle cardiopatie e nefropatie.* SCOGNAMIGLIO. 1898. [Reprint.]
*La Urotropina (Exametilentetramina) e la sua azione terapeutica nelle malattie delle Vie Urinarie.—Sella cura medicamentosa delle malattie delle vie urinarie superiori.* Per il Dottor MARTIN MENDELSOHN. 1898. [Reprint.]

From Docteurs P. HARTENBERG & P. VALENTIN, Paris:

*Revue de Psychologie.* Juin—Août, 1898.

From ROBERT LOVE HOLMES, Glasgow

*Chloroform: its Absolutely Safe Administration.* By ROBERT BELL, M.D. 1898.

From INGRAM BROTHERS, London:

*The English Illustrated Magazine.* July, August, 1898.

From L'INSTITUT DE BIBLIOGRAPHIE, Paris:

*Gazette médicale de Paris.* 3 Septembre, 1898.

From S. KARGER, Berlin:

*Pathologie und pathologischen Anatomie des Centralnervensystems.* Von Dr. ARNOLD PICK. 1898.

From HENRY KIMPTON, London:

*System of Practical Medicine.* By AMERICAN AUTHORS. Edited by ALFRED LEE LOOMIS, M.D., LL.D., and WILLIAM GILMAN THOMPSON, M.D. Vol. III. 1898.
*Operative Gynecology.* By HOWARD A. KELLY, A.B., M.D. Vol. II. 1898.

From B. KÜHN, London:

*Iodoformogen.* By Dr. ERNEST KROMAYER. 1898. [Reprint.]

From ARTHUR LANE, London:

*The Indicator.* July 8, 1898.

From LEA BROTHERS & Co., Philadelphia:

*Medical Climatology.* By S. EDWIN SOLLY, M.D. 1897.

From PAUL LEGENDRE & CIE, Lyon:

*Bulletin de la Société de Chirurgie de Lyon.* Nos. 1, 2. Juin, 1897—Janvier, 1898.

From H. K. LEWIS, London:

*The Extra Pharmacopœia.* By WILLIAM MARTINDALE and W. WYNN WESTCOTT, M.B. Ninth Edition. 1898.
*Notes on Malaria in Connection with Meteorological Conditions at Sierra Leone.* By Surgeon Major E. M. WILSON, C.M.G. Second Edition. 1898.
*Practical Organic Chemistry.* By SAMUEL RIDEAL, D.Sc. Second Edition. 1898.
*Lewis's Library Catalogue.* New Edition. 1898.
*Dwelling Houses.* By W. H. CORFIELD, M.A., M.D. Fourth Edition. 1898.

From THE LIBRARY SUPPLY Co., London:

*The Library World.* Vol. I., No. 1. July, 1898.

From J. P. LIPPINCOTT COMPANY, Philadelphia:

*Lippincott's Medical Dictionary.* 1897.
*System of Diseases of the Eye.* Edited by WILLIAM F. NORRIS, A.M., M.D., and CHARLES A. OLIVER, A.M., M.D. Vol. III. [1898.]
*Genito-Urinary Surgery and Venereal Diseases.* By J. WILLIAM WHITE, M.D., and EDWARD MARTIN, M.D. 1898.

From MACMILLAN AND Co., LIMITED, London:

*Electro-Physiology.* By W. BIEDERMANN. Translated by FRANCES A. WELBY. Two Volumes. 1896–98.

From MORTON & BURT, London:

*Sixty-Third Annual Report of the British Medical Benevolent Fund, for the year 1897.*

From Dr. J. H. MUSSER, Philadelphia:

Symposium on the Pathology of the Diseases of the Cardio-Vascular System. *The Myocardium.* By J. H. MUSSER, M.D., and J. D. STEELE, M.D. [Reprint.] [N.D.]

From YOUNG J. PENTLAND, Edinburgh:

*Renal Growths.* By T. N. KELYNACK, M.D. 1898.
*Edinburgh Medical Journal.* New Series. Vol. III. 1898.

From X. PERROUX ET CIE, Paris:

*La Tuberculose infantile.* No. III. 15 Juin, 1898.

From THE REBMAN PUBLISHING Co., LIMITED, London:

*Archives of the Roentgen Ray.* May, 1898.
*Atlas of Clinical Diagnosis and Internal Diseases.* By Dr. CHRISTFRIED JAKOB. Edited by AUGUSTUS A. ESHNER, M.D. 1898.
*A Text-Book of Diseases of Women.* By CHARLES B. PENROSE, M.D., Ph.D. Second Edition. 1898.
*Health Loss and Gain.* By M. A. CHREIMAN. 1898.
*The Ferment Treatment of Cancer and Tuberculosis.* By HORACE MANDERS, M.D. 1898.

From SMITH, ELDER & Co., London:

*English Sanitary Institutions.* By Sir JOHN SIMON, K.C.B. Second Edition. 1897.
*The Mineral Waters and Health Resorts of Europe.* By HERMANN WEBER, M.D. and F. PARKES WEBER, M.D. 1898.

From T. FISHER UNWIN, London:

*William Stokes.* By his son WILLIAM STOKES. 1898.
*Benjamin Brodie.* By TIMOTHY HOLMES, M.A. 1898.

From Dr. CLARENCE A. VEASEY, Philadelphia:

*A Case of Hypopyon Kerato-Iritis Occurring in a Patient During an Attack of Typhoid Fever.* By CLARENCE A. VEASEY, A.M., M.D. 1898. [Reprint.]

From HUGH M. WILSON, Chicago:

*The Railway Surgeon.* July 12—August 23, 1898.

From J. WRIGHT & Co., Bristol:

*Baby Feeding.* By A DOCTOR. 1898.
*The Ocular Muscles.* By ERNEST E. MADDOX, M.D. 1898.

From K. J. WYSS, Berne:

*Illustrirte Rundschau der Medicinisch-chirurgischen Technik.* 15 August, 1898.

